# Treatment of Class III malocclusion in two phases. functional orthopedics and fixed appliances: A valid option

**DOI:** 10.21142/2523-2754-1403-2026-306

**Published:** 2026-07-10

**Authors:** Herbert M. Orrego C, Luis E. Salazar L

**Affiliations:** 1 Faculty of Dentistry, Postgraduate Program in Orthodontics, Universidad Nacional Mayor de San Marcos. Lima, Peru. herbertorrego@hotmail.com Universidad Nacional Mayor de San Marcos Faculty of Dentistry Postgraduate Program in Orthodontics Universidad Nacional Mayor de San Marcos Lima Peru herbertorrego@hotmail.com; 2 Faculty of Dentistry, Orthodontic Resident, Postgraduate Program in Orthodontics, Universidad Nacional Mayor de San Marcos. Lima, Peru. Email: luisensalaz24@gmail.com Universidad Nacional Mayor de San Marcos Faculty of Dentistry Orthodontic Resident, Postgraduate Program in Orthodontics Universidad Nacional Mayor de San Marcos Lima Peru Email

**Keywords:** class III malocclusion, prognathism, functional appliances, interceptive orthodontics, orthodontic appliances, case report, maloclusión de clase III, pronagtismo, aparatos funcionales, ortodoncia interceptiva, aparatos ortodónticos, informe de caso

## Abstract

The treatment of Class III malocclusions has always represented a challenge for clinicians. This case report presents a patient treated in two stages, initially using functional jaw orthopedics and subsequently fixed orthodontic appliances. The outcomes are described in terms of dental occlusion and soft tissues, highlighting the advantages and disadvantages of this therapeutic approach. We believe that this approach, when the case is properly selected, allows improvement of the patient’s growth pattern, providing a scenario with greater potential for stability of the correction, as well as a more functional and esthetic dental occlusion. Improvement in the soft tissue profile also contributes to emotional well-being in adolescent patients, as observed in the present case.

## RESUMEN

El tratamiento de las maloclusiones de Clase III siempre ha sido un desafío para el profesional. En el presente reporte de caso, se presenta un paciente tratado en dos etapas: inicialmente, se empleó ortopedia funcional de los maxilares y, finalimente, aparatología fija. Se exponen los resultados a nivel de la oclusión dental y tejidos blandos y se indican las ventajas y desventajas de este enfoque. Creemos que este abordaje permite, al seleccionar adecuadamente el caso, mejorar el patrón de crecimiento del paciente, otorgándole un escenario con mayores posibilidades de estabilidad en su corrección, así como una oclusión dental mucho más funcional y estética. La mejora a nivel de los tejidos blandos ocasiona, además, una tranquilidad en la parte emocional de pacientes que atraviesan la adolescencia, como en este caso.

## INTRODUCTION

Class III malocclusions constitute one of the most complex and difficult dentofacial alterations to treat, with a global prevalence ranging from 1% to 19%, depending on ethnic background and diagnostic criteria ^1^. Their etiology is multifactorial and involves skeletal discrepancies such as maxillary retrusion, mandibular protrusion, or a combination of both, in addition to dentoalveolar and functional components ^2^.

Early treatment in growing patients aims to intercept skeletal discrepancies by taking advantage of growth potential to guide the development of the skeletal bases and improve the maxillomandibular relationship ^3^^,^^4^. One of the most frequently employed strategies is two-phase treatment, consisting of an initial orthopedic or functional orthopedic phase intended to modify skeletal growth and establish a more favorable sagittal relationship. Appliances such as facemasks, the bone-anchored maxillary protraction (BAMP) protocol, and functional appliances including bionators, activators, and functional regulators have demonstrated effectiveness in selected patient groups ^5^^,^^6^.

The second, corrective phase with fixed appliances focuses on dental alignment, arch coordination, and occlusal detailing, consolidating the changes achieved during the first phase. Among the advantages of the two-phase approach are early improvement of facial profile and patient self-esteem, reduced complexity of the corrective phase with less need for extractions or surgical procedures, and greater control of mandibular growth during adolescence as a result of prior skeletal modification ^7^. However, disadvantages reported in the literature include longer total treatment duration, which may affect patient cooperation and increase dropout risk, ^1^ higher cost and psychological burden due to prolonged therapy, and the possibility of relapse if residual mandibular growth exceeds the changes achieved during the first phase, potentially leading to the need for orthognathic surgery in adulthood ^8^.

Within this context, the present case report describes the two-phase treatment of a Class III patient using a modified bionator in the initial orthopedic phase, followed by fixed multibracket appliances, highlighting the changes achieved and discussing the advantages and limitations of this approach.

### Case Presentation

An 11-year-3-month-old male patient presented to the orthodontic service of the postgraduate program at the Universidad Nacional Mayor de San Marcos. The parents’ chief complaint was concern regarding their child’s anterior crossbite. The patient had no relevant medical history.

**Figure 1:**
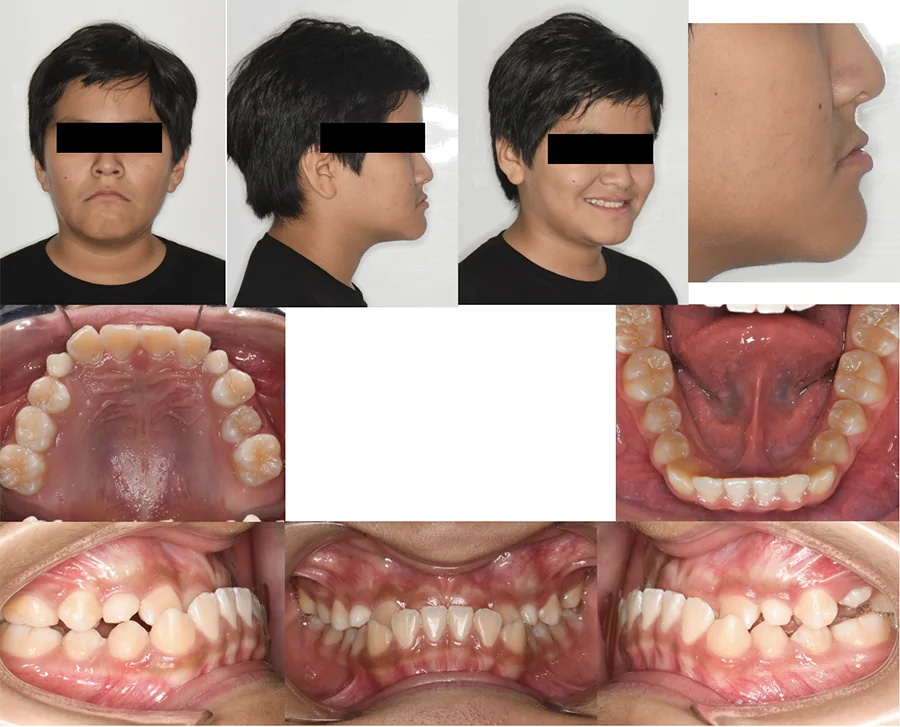
Pretreatment extraoral and intraoral photographs.

Extraoral examination revealed facial symmetry, mesofacial characteristics, increased lower facial third, adequate lip seal, and a concave profile with midfacial depression and prominence of the lips and chin. Intraorally, mixed dentition was observed, with anterior crossbite, Class III molar relationships, non-recordable canine relationships, an overjet of −5 mm, 100% overbite, a 7-mm curve of Spee, and a 0.5-mm deviation of the facial midline to the left (Figure 1).

**Figure 2:**
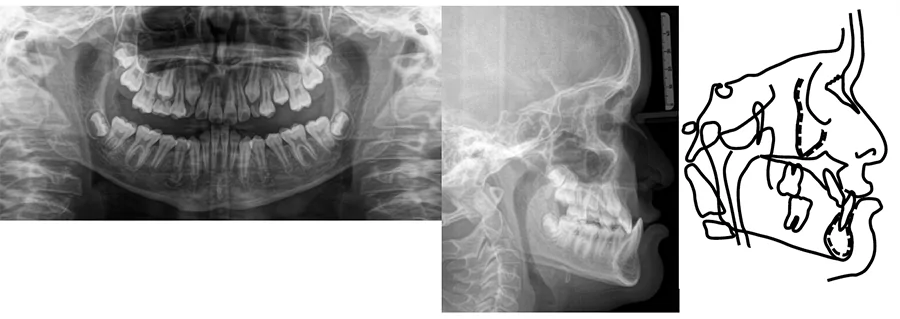
Pretreatment panoramic and lateral cephalometric radiographs, and cephalometric tracing.

Panoramic radiography showed findings consistent with the patient’s biological age. Lateral cephalometric radiography indicated a skeletal Class III relationship due to mandibular protrusion, labially inclined maxillary incisors, lingually inclined mandibular incisors, and a hypodivergent growth tendency. Cervical vertebral maturation analysis revealed a CS2 stage, indicating the beginning of the ascending growth curve (Figure 2).

### Treatment Objectives

The treatment objectives were to improve the skeletal Class III pattern, correct facial imbalance, eliminate the anterior crossbite, correct Class III molar relationships, improve overjet and overbite, level the curve of Spee, establish proper dental intercuspation, and achieve correct functional mandibular excursions.

### Treatment Alternatives

Considering the patient’s age, the objective was to initiate an orthopedic first phase aimed at improving the skeletal relationship. Treatment options included rapid maxillary expansion with facemask therapy followed by fixed appliances; intraoral application of the BAMP protocol using skeletal anchorage followed by multibracket therapy; or functional orthopedic treatment followed by a second fixed phase.

The BAMP protocol was rejected by the family due to its invasive nature, requiring four miniplates. Ultimately, a bionator was selected for the first phase of treatment, followed by preadjusted fixed appliances with MBT prescription for the second phase.

### Treatment Progress

**Figure 3:**
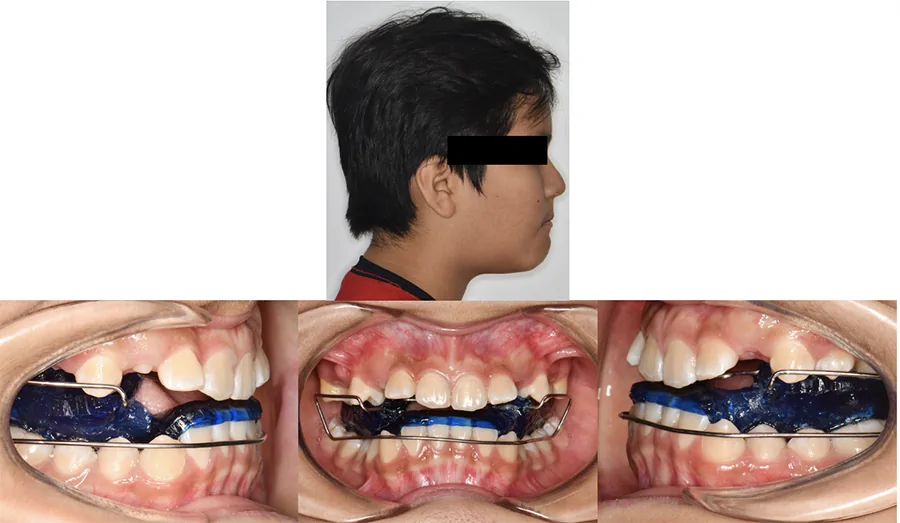
Placement of the bionator appliance. Note the immediate improvement in facial profile.

Treatment began with placement of a bionator appliance with acrylic coverage on the mandibular incisors to function as an inclined plane for the maxillary incisors. Interocclusal acrylic was added to control tooth eruption with the aim of correcting the anterior crossbite and leveling the curve of Spee. The appliance was worn 24 hours per day, removed only for oral hygiene and meals. Improvement in the patient’s facial profile was evident immediately after appliance placement (Figure 3).

At follow-up visits, selective reduction of the interocclusal acrylic was performed to improve dental interdigitation, level the curve of Spee, and correct the anterior crossbite.

**Figure 4:**
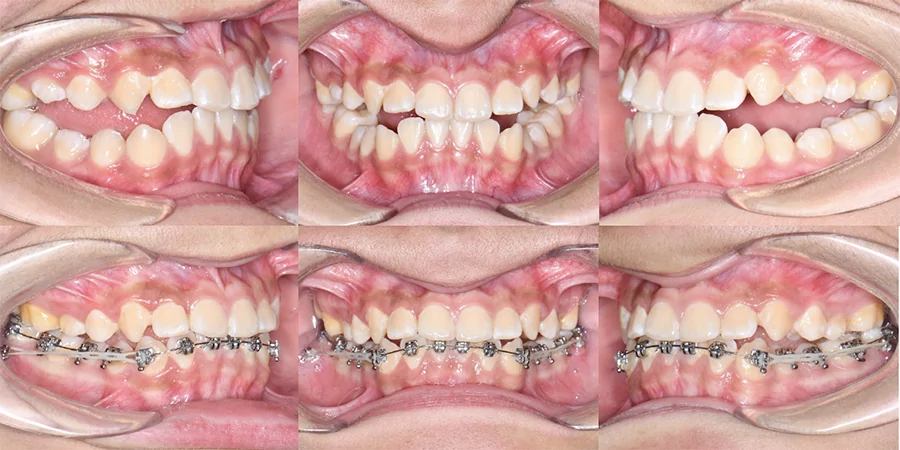
Completion of the bionator phase and initiation of fixed orthodontic appliances.

After 13 months of bionator therapy, improvement of the curve of Spee, correction of the anterior crossbite, and establishment of Class I canine relationships were achieved (Figure 4). Fixed appliances were then placed in the mandibular arch using a 0.022-inch MBT prescription. Two extra-alveolar screws were inserted in the buccal shelf area, and traction of the mandibular arch was initiated to consolidate Class I molar relationships.

**Figure 5:**
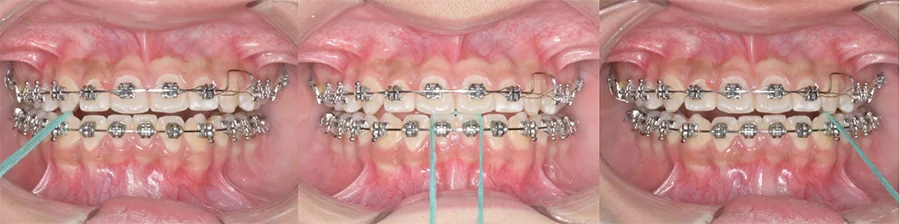
Verification of mandibular excursions. The first phase lasted 13 months and the second phase 11 months, resulting in a total treatment duration of 24 months. Retention consisted of a bonded fixed retainer on the lingual surfaces of the six mandibular anterior teeth and a removable circumferential retainer for the maxillary arch (Figure 6).

Alignment and leveling progressed from 0.014-inch nickel-titanium archwires to 0.019 × 0.025-inch stainless steel archwires, ensuring appropriate arch form and coordination. Finishing bends were performed, and intermaxillary elastics were used for final settling, with verification of mandibular excursions (Figure 5).

**Figure 6:**
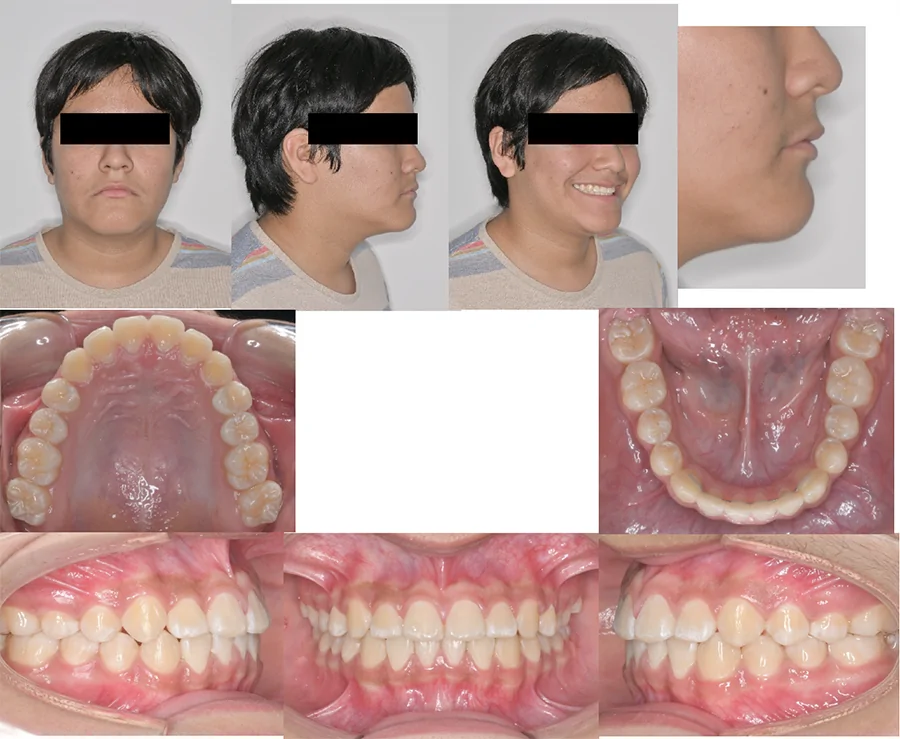
Posttreatment extraoral and intraoral photographs.

**Figure 7:**
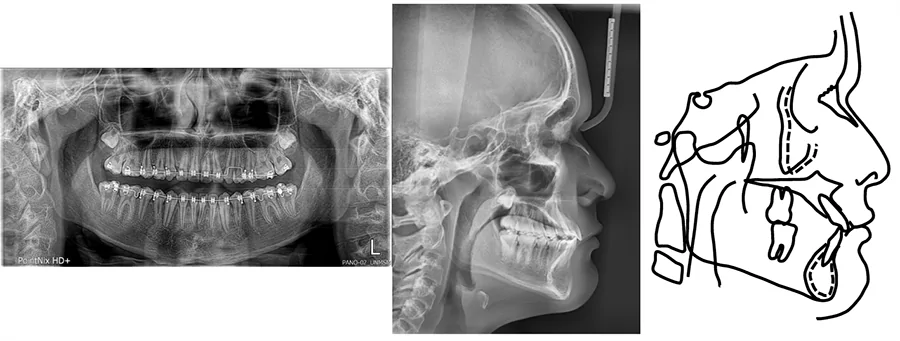
Posttreatment panoramic and lateral cephalometric radiographs, and cephalometric tracing.

Table 1 allows evaluation of the changes achieved through therapy, including improvement in the maxillomandibular relationship, postero-inferior rotation of the mandible, improvement in the inclination of both maxillary and mandibular incisors, and a favorable change in lip position.

**Table 1:** Pretreatment and posttreatment cephalometric values

Measurements	Pretreatment	Posttreatment
SNA (°)	80	81
SNB (°)	86	84
ANB (°)	-6	-3
Wits appraisal (mm)	-14	-4
Maxillomandibular plane angle (°)	11	15
FMA (°)	16	20
MP-SN (°)	21	25
MP-SN (°)	118	128
U1 / A-Pog (mm)	-3	7
IMPA (°)	92	95
L1 / A-Pog (mm)	6	4
Upper lip / E-plane (mm)	-6	3
Lower lip / E-plane (mm	2	3

**Figure 8:**
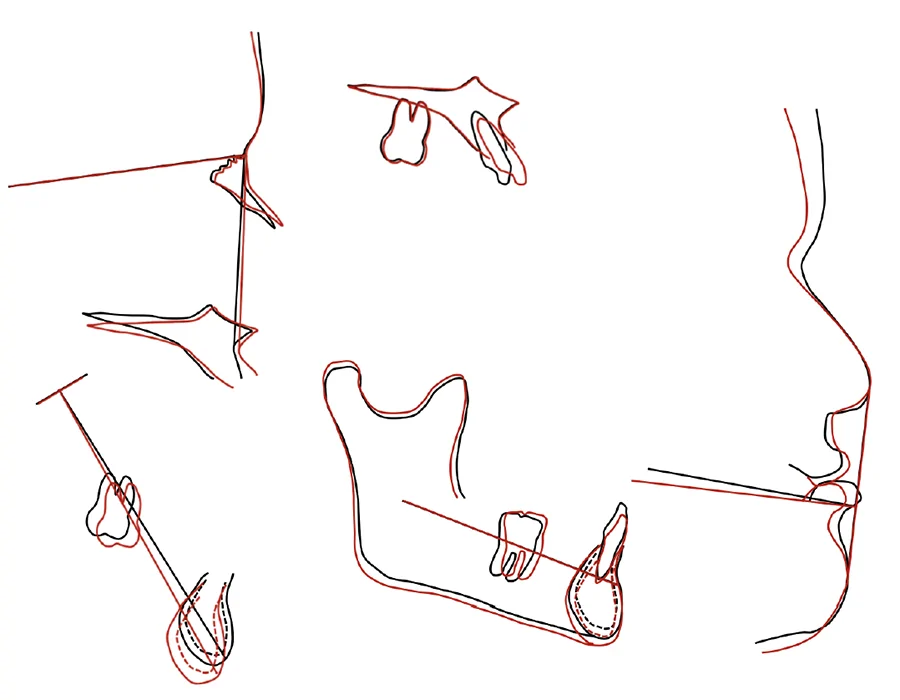
Regional superimpositions in different areas. Black: pretreatment; red: posttreatment.

Analysis of the regional superimpositions (Figure 8) reveals anterior displacement of the maxilla, extrusion of the maxillary molar, downward and backward rotation of the chin, proclination of the maxillary incisor, intrusion of the mandibular incisor, retrusion of the lower lip, and protrusion of the upper lip. These findings reflect the combined skeletal, dentoalveolar, and soft tissue effects achieved through functional orthopedic therapy followed by fixed orthodontic treatment.

## DISCUSSION

Two-phase treatment in growing patients aims to reduce or ideally correct skeletal dysplasia and facilitate the fixed appliance phase, with the goal of avoiding surgical intervention later in life. In Class III therapy, early treatment targeting etiological factors does not necessarily guarantee long-term stability ^9^. Even surgical treatments in patients who have completed growth show relapse rates of approximately 25% of the surgical correction ^10^, with other authors reporting relapse rates of 14-15% of mandibular setback, highlighting the difficulty of long-term prediction ^11^.

Unlike dentofacial orthopedic approaches ^12^, functional orthopedic treatments do not rely on heavy forces but rather on muscular and functional forces of the stomatognathic system. In this case, the constructive bite-set at the most retrusive mandibular position possible-induces a muscular reflex that, when attempting to return the mandible to its original position, exerts traction on the maxilla and its dentoalveolar structures. Because the appliance is bimaxillary, the new dental intercuspation stabilizes the changes achieved. The fixed appliance phase allows for detailed occlusal finishing, promoting a new dental, muscular, and skeletal balance during patient development.

A particularly noteworthy aspect of this case is the marked improvement in facial profile, a commonly reported outcome in functional orthopedic treatments ^13^. This fulfills the primary objectives of orthodontic therapy-morphological, functional, and esthetic improvement-which is highly relevant clinically, as it is readily perceived by patients and their caregivers, thereby increasing treatment adherence.

In this case, both treatment phases were completed within 24 months, indicating that a two-phase approach did not necessarily result in longer overall treatment time. Although modifying skeletal growth is an attractive theoretical concept, clinical outcomes usually reflect a combination of dental and skeletal effects. This should not be considered inadequate, as many orthodontic treatments are inherently compensatory. Early intervention aims to reduce skeletal discrepancies while also producing dental effects, ultimately decreasing the likelihood of surgical treatment later in life.

Current trends, supported by the use of temporary anchorage devices such as miniscrews and miniplates, seek to enhance skeletal effects through less invasive procedures than conventional surgery. These were incorporated during the corrective phase of the present case. Appropriate case selection remains essential, as severe cases may be more appropriately managed with orthognathic surgical approaches, whereas moderate or mild cases may benefit from orthopedic-orthodontic strategies.

## CONCLUSIONS

Treatment planning for patients with Class III malocclusion is always challenging. Considerations such as patient age, severity of the discrepancy in all three planes of space, hereditary factors, and the selected therapeutic approach-orthopedic, surgical, or compensatory-must be carefully evaluated in terms of expected stability.

In the present case, functional and esthetic outcomes were satisfactory, and total treatment time was within an acceptable range. With appropriate case selection and adequate patient cooperation, this treatment option can be successfully applied. Long-term follow-up of Class III cases remains essential to assess stability and potential changes over time.

## Sources of financing:

Nill
